# Impact of elevated atmospheric O_3_ on the actinobacterial community structure and function in the rhizosphere of European beech (*Fagus sylvatica* L.)

**DOI:** 10.3389/fmicb.2014.00036

**Published:** 2014-02-11

**Authors:** Felix Haesler, Alexandra Hagn, Marion Engel, Michael Schloter

**Affiliations:** Research Unit for Environmental Genomics, Helmholtz Zentrum München - German Research Centre for Environmental HealthNeuherberg, Germany

**Keywords:** European beech, Actinobacteria, biocontrol, polyketide synthase gene, ozone

## Abstract

Many bacteria belonging to the phylum of Actinobacteria are known as antagonists against phytpathogenic microbes. This study aimed to analyze the effect of ozone on the actinobacterial community of the rhizosphere of four years old European beech (*Fagus sylvatica* L.) trees during different time points of the vegetation period. Effects of ozone on the total community structure of Actinobacteria were studied based on the analysis of 16S rRNA gene amplicons. In addition effects of the ozone treatment on the diversity of potential biocontrol active Actionobacteria being able to produce antibiotics were characterized by using the type II polyketide synthases (PKS) genes as marker. Season as well as ozone treatments had a significant effect on parts of the actinobacterial rhizosphere community of European beech. However on the basis of the performed analysis, the diversity of Actinobacteria possessing type II PKS genes is neither affected by seasonal changes nor by the ozone treatments, indicating no influence of the investigated treatments on the biocontrol active part of the actinobacterial community.

## Introduction

Background ozone concentrations in the troposphere have doubled in the Northern hemisphere since the industrial revolution and are predicted to remain at high levels in the near future (The Royal Society, 2008). Ozone is a major secondary air pollutant, produced by a complex series of photochemical reactions from primary precursor emissions of nitrogen oxides (NOx) and volatile organic compounds (Ashmore, 2005). Deleterious effects on trees include chronic oxidative plant stress resulting in reduced photosynthesis, foliar injuries and premature leaf loss (Matyssek and Innes, 1999). This leads to an impairment of many metabolic pathways (Olbrich et al., 2005) and reduced primary production due to stomatal closure and damage to leaf mesophyll (King et al., 2005).

Plant derived carbon is the main energy source for soil biological processes and therefore a key factor influencing the community composition of the rhizosphere (Andersen, 2003). The quantity and quality of substances released into the rhizosphere by the plant are known to influence the structure and function of soil-borne microbial communities (e.g., Bais et al., 2006). Since ozone stress may affect carbon input into soils through changes in root physiology and altered root exudation, reduced carbon allocation to the roots due to a reduced photosynthesis could provide less carbon to organisms in the rhizosphere (Andersen et al., 1997), while possible qualitative changes of root exudates might alter nutrient conditions favoring specific rhizosphere organisms. Studying the effect of ozone on microbial groups in the rhizosphere exhibiting specific functions in soil will thus lead to a better understanding about the impact of ozone on microbes and relevant processes in the rhizosphere.

In this respect, plant growth promoting rhizobacteria (PGPR) are of special interest. PGPRs have the potential to stimulate plant growth and/or to actively inhibit performance of plant pathogens and thereby reduce the impact of plant diseases (Compant et al., 2005). Conditions favoring PGPRs in soil therefore have the potential of increasing plant health by reducing the success of pathogens (Janvier et al., 2007). A major group of PGPRs are various members of the phylum Actinobacteria. These microorganisms belong to the Gram-positive bacteria with a high G + C content in their DNA, which are usually able to form branching hyphae at some stage of their development (Goodfellow and Williams, 1983). Members of this group are well known for their ability to produce a wide range of secondary metabolites including commercially important antibiotics such as tetracyclines or cycloheximide (Haesler et al., 2008). The ability of Actinobacteria to inhibit diverse groups of phytopathogens *in vitro* and *in vivo* is a well-documented phenomenon (Paulitz and Belanger, 2001) and their active role in plant rhizospheres has been demonstrated in many studies (Smalla et al., 2001; Billings and Ziegler, 2005; Hjort et al., 2007). A first indication that groups of Actinobacteria might be sensitive to ozone was provided by Dohrmann and Tebbe (2005); they could show a clear change in Actinobacterial community structure in the rhizosphere of the ozone sensitive composite *Sonchus asper*, however no effect could be observed for other plants in the same study. In general, studies on the effect of ozone on the microbial community in rhizosphere of plants are scarce and no analysis has been performed focusing on Actinobacteria in the rhizosphere of woody plants.

A relevant example in this respect is European beech (*Fagus sylvatica* L.). European beech is of great economical importance since it is the most frequently planted deciduous tree species in central European forests (Jung et al., 2005) and also partly detrimental in urban areas, where besides an increase in tropospheric ozone levels also near ground ozone concentrations have increased significantly in the last decades. With respect to ozone, European beech can be considered a sensitive species (Skärby et al., 1998). Yet, interestingly there has been evidence that even though beech trees show a clear reduction of belowground competitiveness under enhanced O_3_, it apparently does not reduce the defense capacity against root pathogens like the oomycetous *Phytophthora citricola*. Luedemann et al. (2005) even postulated an increase in the resistance of European beech trees toward this pathogen in response to increased ozone concentrations in the atmosphere. Even though it is known that O_3_ is capable of eliciting plant responses typically associated with pathogen defense (Matyssek and Innes, 1999), it can also be hypothesized that this stable defense capacity against selected plant pathogens of European beech in the face of other stressors is partly related to a very stable antagonistic biocontrol active microflora in the rhizosphere which inhibits growth and activity of *P. citricola*.

Molecular studies on antagonistic microbial communities often focus on structural rRNA gene analysis of the population rather than on functional antagonistic traits of the organisms. Yet, while phylogeny and expression of phenotypic traits are often closely connected (Berg, 2000; Oda et al., 2003), a lack of correspondence has also been demonstrated for a variety of soil organisms including antibiotics producing streptomycetes (Davelos et al., 2004). Therefore, analyzing changes within groups of possible plant pathogen inhibiting genes, e.g., encoding for antibiotics production, in response to changing environmental factors might be a more conclusive approach to describe the antagonistic population in soil. A large number of antibiotics produced by Actinobacteria are synthesized via type II polyketide synthases (PKS) (Hertweck et al., 2007) making this group of genes an interesting target for culture independent analysis. Among the produced antibiotics and anti-cancer drugs are tetracyclines, anthracyclines, aureolic acids and many more. Wawrik et al. (2005) developed primers for terminal restriction fragment length polymorphism (T-RFLP) analysis of these genes in soil and conducted an ecological study comparing soils from New Jersey to Uzbekistan (Wawrik et al., 2007). However, the applicability of these primers for studies within one ecosystem, where less variability is to be expected, has not been tested so far.

This study aimed to analyze the effect of ozone on the actinobacterial community of the rhizosphere of four-year old European beeches during different times of one year (spring, summer and autumn). Effects of ozone on Actinobacteria were studied on the structural level of the community (16S rRNA gene), as well as on the functional level using primers targeting type II PKS genes by fingerprinting and clone library analysis.

## Materials and methods

### Experimental setup and sampling

In 2006, 36 pots (14 L) were planted with three four-year old saplings of European beech each and incubated outside. Beech seedlings were obtained from a nursery (Bayr. Staatsforsten, Laufen, Germany). The soil was taken from the Ah-B horizont of mixed beech/spruce stand in the “Eurasburger Forst” near Augsburg, Germany (11° 5′ E and 48°18′ N). This soil has been characterized as a podsolic para-brown soil (orthic luvisol). Soil pH (H_2_O) was 3.9 and C/N analyses result in 6.4% of total carbon, 0.3% of total nitrogen and a C/N ratio of 19.9. The soil texture was: 41% sand, 36% silt and 23% clay. (Kreutzer et al., 1991).

In spring 2009 before the start of the vegetation period the pots were transferred in climate controlled greenhouse chambers at the Helmholtz Zentrum München. Half of the pots were exposed to ambient O_3_ conditions (natural O_3_ levels outside the greenhouse ranging from 20 to 80 ppb), the other half to twice ambient O_3_ conditions (restricted to <150 ppb). Therefore pots were placed into two tents volume about 7000 l) built of transparent plastic foil (ethylene-tetrafluorethylene ETFE, film thickness 80 mm, Koch Membranen GmbH, Rimsting, Germany), to separate the plants from the outer greenhouse atmosphere and incubated under the respective ozone concentrations. Ozone concentrations were measured continuously using an O_3_-analyser (Columbia Scientific Industries, Austin, USA). Fumigation was performed with an O_3_-generator (Fischer, Meckenheim, Germany) via a compressor. Ozone concentrations were adjusted automatically every hour. Relative humidity was adjusted in all pots to outside conditions and only natural light was used. Irrigation was carried out automatically, starting out with 200 mL of demineralized water every 56 h in spring and adjusting regularly to changing water demands due to increased temperature and plant performance. 150 mL of double strength Hoagland solution (Hoagland and Arnon, 1950) were applied as fertilizer in April, June and August.

Sampling took place at three different time points throughout of the vegetation period (bud break in May, full leaf development in July and senescence in September. At each time point six pots for each of the two treatment combinations (1 × O_3_; 2 × O_3_) were harvested and treated as true replicates. Roots were cleaned from loosely adhering soil by hand. Soil tightly attached on the root surface was rinsed using phosphate buffer solution, and centrifuged at 8000 × g for 10 min. The pellet was collected as rhizosphere soil. Samples from one pot (three plants) were pooled to minimize the effect of genetic variation between different beech trees, resulting in approximately 10 g of rhizosphere soil per pot and stored in plastic bags at −80°C until further processed.

### DNA extraction

Environmental DNA was extracted using the Fast Spin DNA Extraction Kit for Soil (MP Biomedicals, Eschwege, Germany) according to the manufacturer's instructions with modifications. 0.5 g of homogenized soil was used. The original protocol was modified by adding two washing steps of the silica binding matrix using 5.5 M guanidine thiocyanate solution to remove inhibitory substances. DNA extractions from soil samples were performed in duplicates. The amount of DNA was estimated by Nanodrop spectrophotometer (NanoDrop Technologies, Wilmington, USA).

### PCR amplification

To evaluate the diversity of Actinobacteria in beech rhizosphere the following Actinobacteria specific 16S rRNA gene primers were used: as forward primer S-C-Act-235a-S-20, 5′-CGCGGCCTATCAGCTTGTTG-3′ (Stach et al., 2003) and as reverse primer Act-1360, 5′-CTGATCTGCGATTACTAGCGACTCC-3′ (McVeigh et al., 1996). Amplification was performed as follows: each 50 μ L reaction contained 1x buffer (Gibco BRL, Karlsruhe, Germany), 0.2 μ M of each primer (Thermo Hybaid, Ulm, Germany), 2 mM MgCl_2_ (Gibco BRL, Karlsruhe, Germany), 0.2 mM of each dNTP (MBI Fermentas, St. Leon-Rot, Germany), 5% DMSO, 0.3% BSA, 20 ng of template DNA and 2.5 U *Taq* polymerase (Gibco BRL, Karlsruhe, Germany). A hot start was applied with a denaturation at 95°C for 5 min followed by 30 cycles of 95°C for 45 s, 72°C for 1 min and 45 s and a final elongation at 72°C for 5 min.

For a culture independent functional analysis of microbial communities the type II PKS specific primer pair 540f, 5′-GGXTGCACSTCXGGXMTSGAC-3′, and 1100r, 5′-CCGATSGCXCCSAGXGAGTG-3′, (Wawrik et al., 2005) was used (X = inosine). For amplification, a 50 μ L PCR reaction consisted of 1x buffer (Gibco BRL, Karlsruhe, Germany), 0.4 μ M of each primer (Thermo Hybaid, Ulm, Germany), 2 mM MgCl_2_ (Gibco BRL, Karlsruhe, Germany), 0.2 mM of each dNTP (MBI Fermentas, St. Leon- Rot, Germany), 5% DMSO, 0.3% BSA, 20 ng of template DNA and 5 U Taq polymerase (Gibco BRL, Karlsruhe, Germany). The cycle program was initiated with a hot start for 5 min at 95°C followed by 35 cycles of 95°C for 1 min, 68°C for 1 min and 72°C for 45 s and a final elongation at 72°C for 10 min.

### TRFLP analysis

PCR amplification was performed with primers which were Cy5 fluorescently labeled at the 5′-terminal end. For 16S rRNA gene amplification primer S-C-Act-235a-S-20 and for PKS type II gene amplification primer 540f was labeled. For each DNA extract PCRs were repeated three times, the products were subsequently pooled and purified with PCR Purification Kit (Qiagen, Hilden, Germany). A double digestion with enzymes *MboI* (New England Biolabs, Frankfurt am Main, Germany) and *Fau*I (SibEnzyme, Zweibrücken, Germany) was performed for actinobacterial 16S rRNA gene TRFLP analysis. For the first digestion reaction, a total volume of 10 μ L contained 1x NEBuffer 1 (New England Biolabs, Frankfurt am Main, Germany), 2.5 U of *Mbo*I and 100 ng of pooled PCR products. The digestion mixture was incubated for 16 h at 37°C. Then, 10 μ L of *Fau*I solution containing 1 U of enzyme in 1x NEBuffer 1 was added. This reaction mixture was incubated at 55°C for 16 h, subsequently heated to 65°C for 20 min to inactivate the enzymes and cleaned using the Minelute PCR Purification Kit (Qiagen, Hilden, Germany). For the analysis of PKS type II genes, 100 ng of the PCR products were digested with 20 U *Hha*I (New England Biolabs, Frankfurt am Main, Germany) in a total of 20 μ L of 1x NEBuffer 4 (New England Biolabs, Frankfurt am Main, Germany) supplemented with 2 μ g BSA. DNA was digested for 18 h at 37°C followed by an enzyme inactivation at 65°C for 20 min. The reaction mix was purified using the Minelute PCR Purification Kit (Qiagen, Hilden, Germany).

For detection of labeled fragments 2.5 μ L of the purified digestion reaction was mixed with 0.25 μ L GenomeLab DNA Size Standard 600 (Beckman Coulter GmbH, Krefeld, Germany) and 27.25 μ L SLS buffer (Beckman Coulter GmbH, Krefeld, Germany). Separation of the fragments was conducted using a CEQ 2000 XL sequencer (Beckman Coulter GmbH, Krefeld, Germany). Each reaction was run three times on different capillaries to minimize capillary effects. One representative profile was taken for each sample for further analysis. To analyze peak profiles the CEQ 8000 Genetic Analysis System software version 8.0.52 (Beckman Coulter GmbH, Unterschleißheim, Germany) was used. Peak recognition was checked and edited manually to include all peaks within a profile. Peak heights were expressed relative to total peak height within a sample and all peaks below 0.5% of the total peak height within a sample were excluded from the analysis. Mean values were calculated for each peak from the duplicate DNA extractions for each soil sample.

### Clone library analysis

To identify major peaks of the TRFLP profiles a reference data basis was generated for 16S rRNA and PKS type II genes based on clone libraries using DNA extracted from the 1 × O_3_ treatment taken during full leaf development in July, where the most pronounced influence of the plant on the rhizosphere microflora was expected due to highest assimilation and exudation rates. The PCR products were cloned into a pCR® 2.1-TOPO® vector with the TA Cloning® Kit (Invitrogen, Karlsruhe, Germany) according to the manufacturer's instructions. Selection of positive clones was done by standard blue-white screening (Sambrook et al., 1989). Colonies were grown over night in 2–5 mL LB broth containing 50 μ g/mL kanamycin and were used for plasmid extraction according to Bimboim & Doly (11). Plasmids containing inserts of the correct size were selected after digestion with EcoRI (MBI Fermentas, St. Leon-Rot, Germany) and sequenced. For the 16S rRNA gene library 56 clones and for the PKS type II library 54 clones were analyzed respectively. Sequencing was carried out using the BigDye Terminator Kit v3.1 (Applied Biosystems, Foster City, USA) and reactions were performed according to manufacturer's instructions on an ABI 3730 sequencer (Applied Biosystems, Foster City, USA).

16S rRNA gene sequences of the clone banks were classified using the higher-order bacterial taxonomy implemented in the Ribosomal Database Project II Release 9.54 (Cole et al., 2005) naïve Bayesian rRNA classifier (http://rdp.cme.msu.edu/) (Wang et al., 2007). For PKS type II sequences, a phylogenetic tree was calculated from protein sequences using the maximum-likelihood algorithm implemented in ARB (http://www.arb-home.de) after aligning the sequences with the ARB Fast Aligner tool. For testing the robustness of tree topology, trees were additionally reconstructed using parsimony (phylip protein sequence parsimony method—ProtPars, implemented in ARB) and neighbor joining methods (correction: PAM matrix) implemented in ARB. Actinobacterial 16S rRNA gene sequences were submitted to GenBank database with the accession numbers EU138966-EU139021 and PKS type II gene sequences with the number EU138915-EU138965.

### Statistical analysis

Two indices, a moving window analysis (MWA) of the actinobacterial community based on a % change value matrix (calculated from a Pearson product-moment correlation coefficient matrix) expressed as the rate of change between two consecutive harvests (Δ_*t*_), and a Pareto-Lorenz (PL) curve as introduced by Marzorati et al. (2008), were calculated to describe the T-RFLP profiles. To do this, the respective peaks are ranked from high abundance to low abundance, based on their peak heights. The cumulative normalized number of bands can be used as *x*-axis, and their respective cumulative normalized intensities represent the *y*-axis. The more the PL curve deviates from the 45° diagonal (the theoretical perfect evenness line), the less evenness can be observed in the structure of the studied community. To compare curves from different samples with each other, the *y*-axis projection of their respective intercepts with the vertical 20% *x*-axis line (PL20) can be scored (Wittebolle et al., 2008).

While MWAΔ_*t*_ describes the rate of change of a population, PL20 can be interpreted as an indication of the dominance of the most common groups of a population and therefore the evenness observed in the community structure. Univariate ANOVA was used to compare the values of different treatments utilizing S-Plus package Version 6.2 (Insightful Corp., Seattle, USA).

Non-metric multidimensional scaling (NMS) on the basis of Euclidean distance measure was used as an unconstrained ordination method to visualize patterns for multivariate data sets utilizing PC-ORD version 5.0 (MjM Software, Gleneden Beach, USA). The best dimensionality for the data sets was assessed by comparing stress values of 250 runs performed for 1-D to 6-D solutions. Additional dimensions were considered useful if they reduced the final stress by five or more. For all data sets 2-D solutions fitted this criterion. To evaluate whether NMS extracted stronger axes than expected by chance this procedure was repeated with randomized versions of the data sets and compared with the real data (Monte Carlo test). For all solutions the *p*-value was *p* < 0.01. For final solutions a maximum of 500 iterations was set using a stability criterion of <0.0000001 for the last 10 iterations (McCune and Grace, 2002).

To test for differences in composition and relative abundance of the multivariate data between samples from different treatments or groups non-parametric multivariate analysis of variance (PerMANOVA) was used (Anderson, 2001). For each term in the analysis, 4999 permutations of raw data units were done to obtain *p*-values. Individual pair-wise multiple comparisons by permutation were performed for factors showing significant differences (4999 permutations). In cases where there were not enough permutations possible to get a reasonable test 4999 Monte Carlo samples were drawn from the theoretical asymptotic permutation distribution Analyses were carried out using the FORTRAN program PerMANOVA (http://www.stat.auckland.ac.nz/~mja/Programs.htm).

To contrast the abundance of T-RFs across different groups of samples, indicator species analysis was performed according to Dufrêne and Legendre (1997) as implemented in PC-ORD version 5.0 (MjM Software, Gleneden Beach, USA).

## Results

### Plant response to the ozone treatment

Whereas a significant increase of the plant biomass over the vegetation period was visible the ozone treatment did not influence the development of the above ground plant biomass. For the below ground biomass in contrast besides the effect of the sampling time point during the vegetation period, also an influence of the ozone was visible, resulting in a higher root biomass mainly in July in the pots treated with double ozone concentrations compared to the pots where only ambient ozone had been applied (data not shown). This indicates at least for the belowground biomass pronounced changes in the ecophysiology of the plants in response to the ozone application.

### Influence of increased ozone levels on actinobacterial community structure in the rhizosphere

#### Changes in diversity of actinobacteria over the vegetation period and in response to ozone

Partial actinobacterial 16S rRNA genes were successfully amplified from all soil samples and reproducible T-RFLP profiles could be obtained from environmental DNA. In most cases profiles of replicates for each treatment produced very similar patterns of peak intensities (coefficient of variation of ~6.5% for the major peaks). Thus, the observed differences in the profiles of treatments can be attributed to effects of the factors season and ozone elevation.

From the 36 samples included in the analysis of actinobacterial 16S rRNA genes, 39 different T-RFs were identified after relativization and removal of background noise. When comparing the average peak intensities for each treatment combination, the actinobacterial rhizosphere community was dominated by few major groups. The mean PL20 for all samples was 87.2 (±1.6) and did neither differ significantly between ambient (87.6 ± 0.8) and ozone treated samples (86.8 ± 2.1) nor between spring (88.4 ± 1.3), summer (86.8 ± 1.7) and autumn (86.4 ± 1.3) samples. Even though the relative evenness of the samples was not influenced by any of the factors, the quality of the profiles changed over time. The dissimilarity between spring and summer samples (Δ_*t*(spring/summer)_ = 13.4 ± 3.1 for ambient and 11.7 ± 4.4 for ozone treated plants) was higher than between summer and autumn (Δ_*t*(summer/autumn)_ = 4.2 ± 1.5 for ambient and 5.7 ± 2.1 for ozone treated plants). This difference was statistically significant (*p* < 0.0001) for the different sampling times and not significantly different between the two treatments.

**Figure 1:**
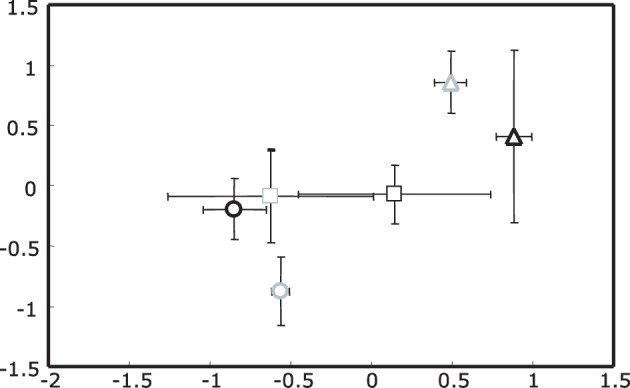
**Non-metric Multidimensional Scaling (NMS) plot of actinobacterial 16S rRNA gene T-RFLP profiles for rhizosphere DNA**. Upright triangles indicate spring, circles summer and squares autumn samples. Ozone treated samples are shown in gray, while controls are black. Stress value was 4.48 given according to Kruskal's stress formula 1 multiplied by 100.

For 16S rRNA gene profiles a two dimensional plot captured most of the variance in the T-RFLP profiles, with the first two dimensions containing 63.7 and 35.1% of the information in the analytical data set respectively (cumulative = 98.8%). The most dominant effect seen in the NMS plot was for samples collected in spring (upright triangles, Figure 1); this effect was independent of the ozone treatment. Separation of summer and autumn samples was not as pronounced with the exception of ozone treated summer samples which formed a distinct cluster.

These observations were in agreement with the results of a non-parametric multivariate analysis of variance (PerMANOVA) and “a posterior” performed multiple pair-wise comparisons. With PerMANOVA, the effects of factors season and treatment were statistically significant (with *P* = 0.0002 and *P* = 0.0288 respectively). When performing a multiple pair-wise comparison between the three levels of the factor season, it was apparent that all seasons were significantly different from each other (spring vs. summer *P* = 0.0002, spring vs. autumn *P* = 0.0006 and summer vs. autumn *P* = 0.0042). Since there was a significant interaction between factors season and treatment (*P* = 0.0238), the effect of the treatment was analyzed separately within each level of the factor season, indicating that the separation of rhizosphere samples from ozone treated plants was only statistically significant in summer (*P* = 0.0026).

#### Identification of main responders

In order to find T-RFs responsible for the separation of different groups in the NMS plots of actinobacterial 16S rRNA genes, an indicator species analysis was performed. Groups chosen for a detailed analysis were, first, spring samples vs. autumn and summer samples and, secondly, a contrasting of the ozone treatment for samples harvested in summer. T-RFs with a significant result in the indicator species analysis were further analyzed by means of a permutation based univariate ANOVA with “a posterior” multiple pair-wise comparison (Table 1).

**Table 1:** **Results of indicator species analysis in combination with univariate ANOVAs based on selected t-RFs of the 16S rRNA amplicon profiles**.

**(A) Indicator species analysis contrasting spring vs. summer and autumn samples**											
**T-RF**	**IG**	**Mean relative peak height within groups (%)**						**PerMANOVA**	**Pair-wise comparisons**		
		***sp***	**SD**	***su***	**SD**	***au***	**SD**	**[*P*-value]**	***sp* vs. *su***	***sp* vs. *au***	***su* vs. *au***
69	sp	10.9	1.5	8.1	1.0	8.0	1.2	0.0002	0.0002	0.0002	ns
**102**	**su**	**12.3**	**3.4**	**22.4**	**2.9**	**18.1**	**3.9**	**0.0002**	**0.0002**	**0.0010**	**0.0064**
162	sp	14.6	1.6	12.2	1.6	13.0	1.5	0.0016	0.0022	0.0170	ns
226	sp	3.1	0.4	2.3	0.6	2.1	0.9	0.0014	0.0004	0.0010	ns
367	sp	2.6	0.4	1.9	0.5	1.9	0.4	0.0002	0.0006	0.0004	ns
378	sp	1.0	0.3	0.7	0.2	0.9	0.2	0.0022	0.0002	ns	0.0180
380	sp	6.6	0.8	4.3	0.6	5.3	1.1	0.0002	0.0002	0.0026	0.0136
**(B) Indicator species analysis contrasting ambient vs. O3 samples in summer**.											
**T-RF**	**IG**	**Mean relative peak height within groups (%)**						**PerMANOVA**			
		***am***	**SD**	***O_3_***	**SD**			**[*P*-value]**			
160	O3	1.0	0.1	1.4	0.1			0.0040			
226	O3	1.9	0.3	2.7	0.5			0.0100			
411	am	1.3	0.3	0.5	0.2			0.0040			
**579**	**am**	**14.8**	**2.5**	**8.9**	**1.8**			**0.0010**			

*Values in bold refer to dominant T-RFs. Selected indicator peaks are shown. Values in bold highlight major peaks that were most likely to be responsible for the separation of the groups. P-values for univariate ANOVA were obtained by 4999 permutations for each peak. P-values in italics were obtained using 4999 Monte Carlo samples from the asymptotic permutation distribution. IG, contrast group indicating the group with the highest abundance of the peak; sp, spring; su, summer; au, autumn; am, ambient; SD, standard deviation*.

**Figure 2:**
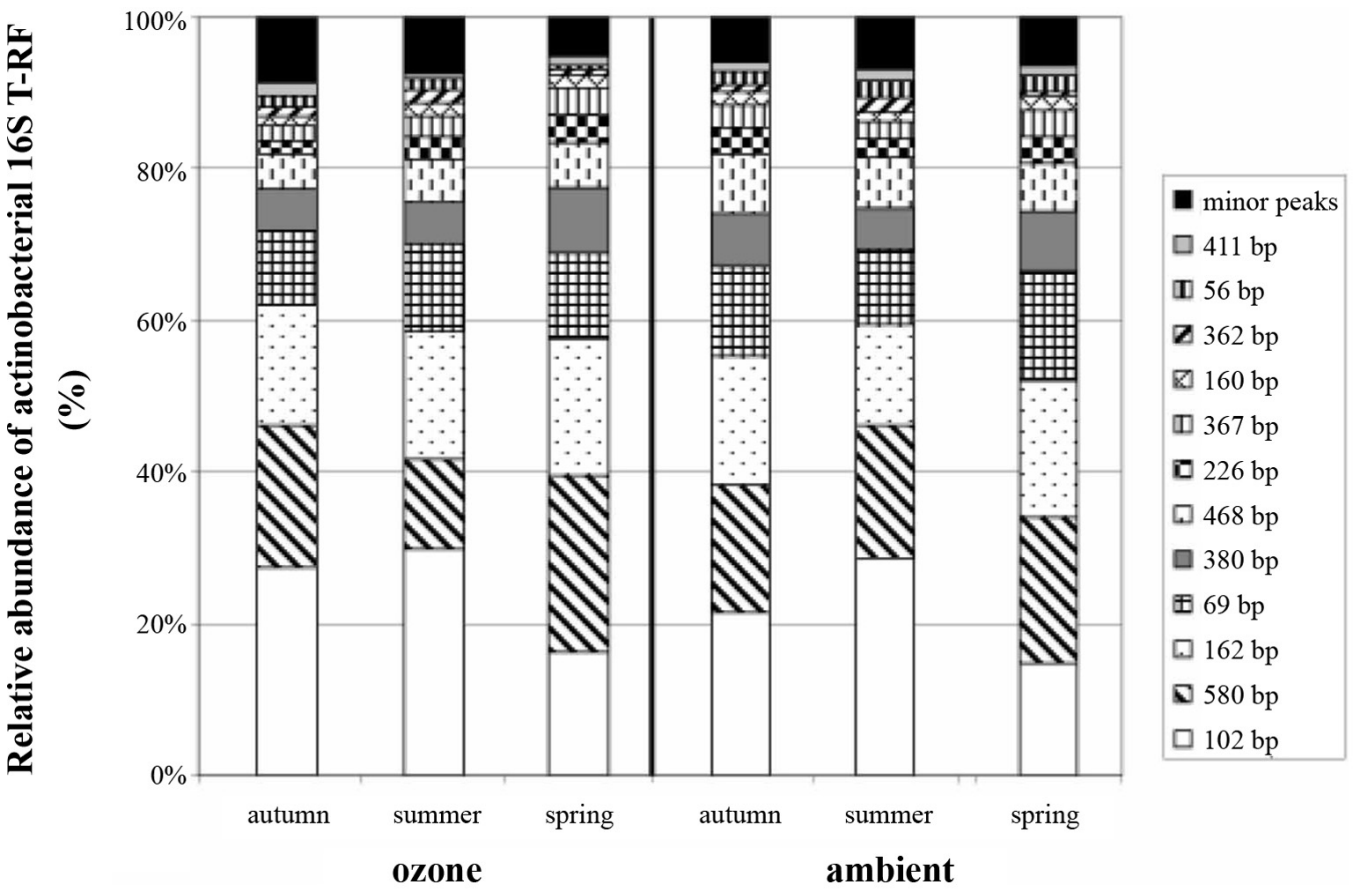
**Average relative abundance of actinobacterial 16S rRNA gene T-RFs from replicate soil samples**. Peak size is given in base pairs, and relative abundance of T-RFs is given as percentage of total peak height.

Of the T-RFs tested positive for an indication of spring samples, T-RF 102 stands out as the most dominant peak (Figure 2). It can be considered as negative indicator for spring since the average relative peak height of this T-RF almost doubled from 12.3% in spring to 22.4% in summer, followed by a small reduction toward autumn to 18.1%. These differences were statistically significant between all groups. Other major T-RFs like peaks 69, 162, 226, and 380 were positive indicators of spring. Yet, the observed differences in relative abundance for those peaks were generally rather small and should therefore not be over interpreted. Still it was obvious that a change in the overall composition of the actinobacterial rhizosphere community took place throughout the year.

When we tried to figure out the T-RFs which were responsible for the separation of T-RFLP profiles in response to the ozone fumigation in summer, most of the T-RFs identified were only very minor peaks, with the exception of T-RF 579. The phyla represented by this peak were less abundant in the rhizosphere of ozone treated plants. The ozone effect was statistically significant, but could only be seen at the summer harvest (Table 1B). Therefore, the separation of ozone treated samples in the NMS plot during summer was very likely due to a change in the abundance of this peak. T-RF 411 was a second good, yet only minor, indicator showing significant influence of the treatment.

#### Phylogeny main responders

The established actinobacterial 16S rRNA gene clone bank confirmed the high specificity of the chosen primer pair S-C-Act-235-a-S-20/Act1360. Out of the 56 clones all could be assigned to the phylum Actinobacteria, 21 of which had to be categorized as unclassified (showing less than 95% probability of belonging to a genus). The remaining clones were dominated by sequences belonging to the family Catenulisporaceae (19 clones of the genus *Actinospica* and 4 of the genus *Catenulispora*). Another large fraction of the clones was related to *Mycobacterium*; for each of the genera *Nocardioides*, *Pseudonocardia*, *Rhodococcus* and *Terrabacter* one clone was detected

When comparing the fragment sizes of the indicator T-RFs with peaks obtained from the clone library, it was possible to identify actinobacterial genera which were influenced by the sampling time point. Organisms belonging to the family Catenulisporaceae (genera *Actinospica* and *Catenulispora*) formed were very likely to be responsible for the T-RF with the fragment size of 102 bp. While all clones belonging to the genus *Actinospica* had the same size, the four *Catenulispora* clones produced three different T-RF fragment sizes (Table 2).

**Table 2:** **T-RF sizes of the amplicons of 16S rRNA gene fragments from clones and pure cultures after double digest (*MboI/FauI*)**.

Clone no./ strain^*^	**Genus**	**Genus**	**Enzyme**	**Expected fragment size [bp]**	**Actual fragment size [bp]**
A15	*Actinospica*	Catenulisporaceae	*FauI*	106	102
A7	*Catenulispora*	Catenulisporaceae	*FauI*	106	102
A19	*Catenulispora*	Catenulisporaceae	*FauI*	366	362
A37	*Catenulispora*	Catenulisporaceae	*FauI*	388	381
A2	*Mycobacterium*	Mycobacteriaceae	*FauI*	230	226
A6	*Mycobacterium*	Mycobacteriaceae	*FauI*	378	371
A27	*Mycobacterium*	Mycobacteriaceae	*FauI*	375	367
A49	*Nocardioides*	Nocardioidaceae	*MboI*	154	149
A13	*Pseudonocardia*	Pseudonocardineae	*FauI*	476	471
A17	*Rhodococcus*	Nocardiaceae	*FauI*	473	464
A39	*Terrabacter*	Intrasporangiaceae	*MboI*	596	596
PT-1	*Kitasatospora*	Streptomycetaceae	*MboI*	415	411
PT-7	*Streptomyces*	Streptomycetaceae	–	–	409

* *A, clone from actinobacterial 16S rRNA gene amplicon library; PT, 16S rRNA gene of actinobacterial isolates (see Haesler et al., 2008)*.

None of the classified clones from the 16S rRNA gene library corresponded to either T-RF 579 or 411, which showed a clear response the ozone fumigation. Yet, a group of unclassified clones were shown to have a T-RF of 578 bp (data not shown). Also, T-RFs from actinobacterial isolates from the same soil belonging to the genera *Kitasatospora* and *Streptomyces* gave signals at 411 and 409 bp respectively (Table 2) and were therefore might represented by this peak in the profiles.

### Influence of increased ozone levels on actinobacterial community function in the rhizosphere

#### Changes in diversity of actinobacteria harboring the PKS II gene over the vegetation period and in response to ozone

**Figure 3:**
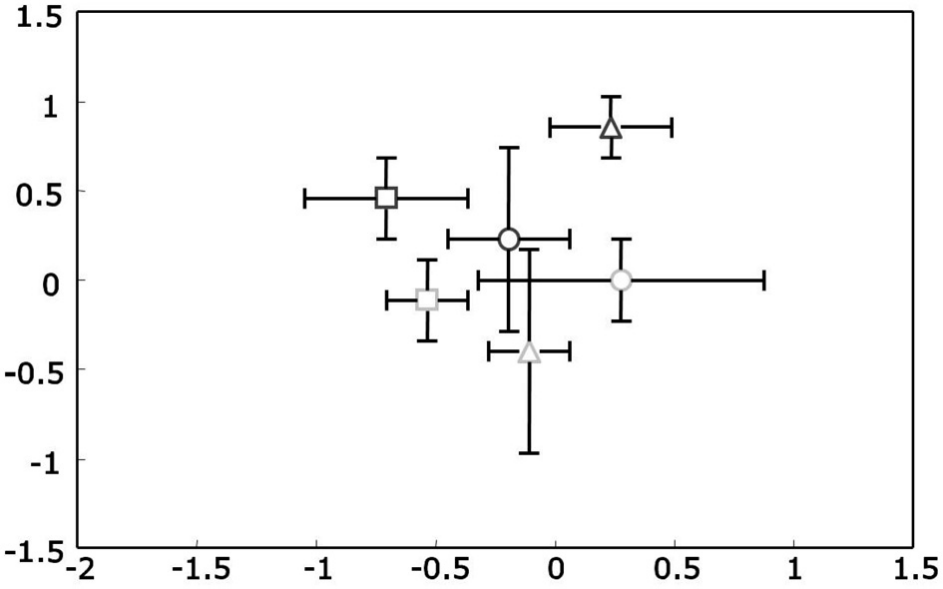
**Non-metric Multidimensional Scaling (NMS) plots of PKS Type II t-RFLP profiles for rhizosphere DNA**. Upright triangles indicate spring, circles summer and squares autumn samples. Ozone treated samples are shown in gray, while controls are black. Stress value was 4.96 given according to Kruskal's stress formula 1 multiplied by 100.

Like for 16S rRNA genes also for PKS type II genes DNA from all samples was successfully amplified and the obtained TRFLP profiles from replicates were highly comparable. Overall the T-RFLP profiles were less diverse compared to actinobacterial 16S rDNA profiles, as was expected, and were also strongly dominated by a few major peaks. An overall analysis of all samples based on NMS plots did not show any response of the PKS II harboring Actinobacteria to the treatments season and ozone (Figure 3). Therefore to study changes in the diversity of these antibiotic producing genes two separate analyses were performed. One analysis was done from all ambient treated samples for each season (a total of 18 samples). The other analysis was performed with the 12 summer samples to analyze the effects of the ozone treatment. The summer harvest was chosen based on the observations that the clearest separation of actinobacterial 16S rRNA gene T-RFLP profiles was seen for this season. After relativization and removal of background noise 26 (for seasonal analyses) and 28 (for treatment analyses) T-RFs were included in the matrices respectively.

The mean PL20 for all samples was even higher than for the 16S rDNA profiles at 92.2 ± 3.8. There were no statistically significant differences neither for the factor season nor the factor ozone (when analyzed for the summer sampling). The dissimilarity between ambient spring and summer samples (Δ_*t*(spring/summer)_ = 7.2 ± 1.6) did not differ significantly from the dissimilarity between summer and autumn (Δ_*t*(summer/autumn)_ = 7.0 ± 3.2). Additionally, dissimilarity between ambient and ozone treated summer samples was in the same range at Δ_*t*(ambient/ozone)_ = 7.5 ± 2.3 indicating no influence of any of the factors. These observations were verified with PerMANOVA and for all factors, season, and ozone, no statistically significant differences were observed.

#### Diversity of actinobacteria harboring the PKS II gene

**Figure 4:**
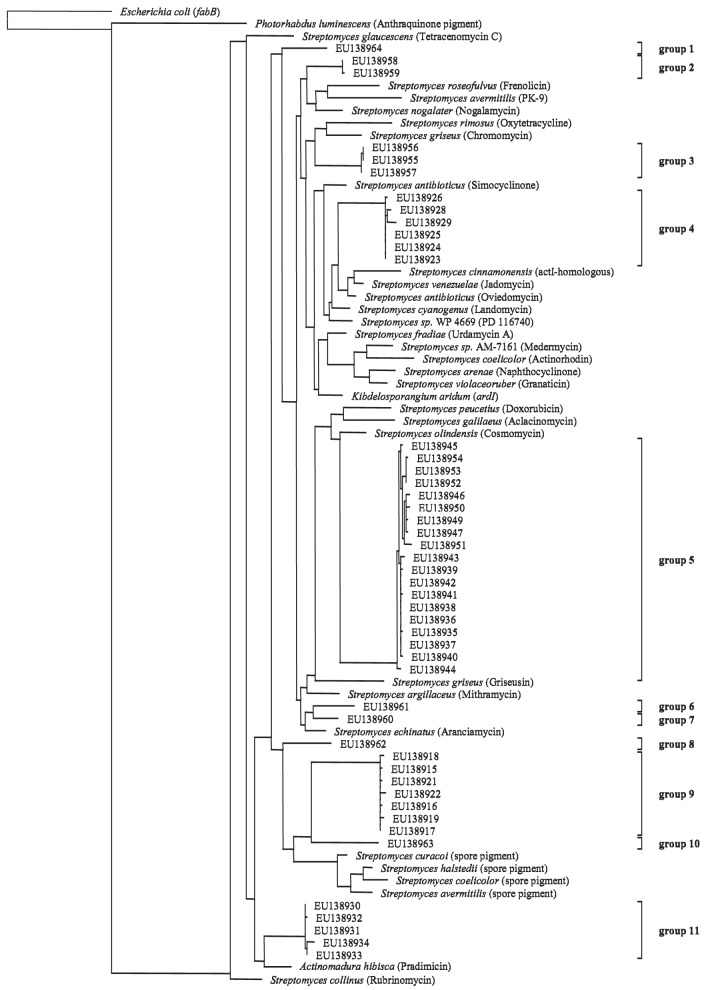
**Maximum-likelihood tree based on partial PKS type II protein sequences (185 amino acid positions) from cultured polyketide producers with known PKS Type II sequences (NCBI database) and sequences from beech rhizosphere clone library (accession numbers indicated)**. Tree topology was supported by parsimony and neighbor-joining methods (data not shown). Products of the reference PKS systems are given in parentheses. Groups are assigned on the basis of 95% similarity of the aminoacid sequences. The scale bar indicates 10% dissimilarity in aminoacid sequences.

Although no clear response of the Actinobacteria harboring the PKS II gene in response to the factors season and ozone was visible, a clone library was constructed to do a phylogenetic identification of major groups of Actinobacteria harboring the PKS II gene. Out of the 54 selected clones 51 sequences had polyketide type II KS-domains as closest hits when submitted to a database search (blastx). The remaining three did not show any homology to known proteins. The nucleic acid codes of all obtained sequences were translated into protein sequences and aligned. Upon translation it was discovered that two of the sequences included internal stop codons. These sequences were considered to be pseudogenes and were therefore excluded from the phylogenetic analysis (accession numbers: EU138920 and EU138948). A maximum-likelihood tree was calculated for the remaining clones and 32 reference protein sequences (Figure 4). References were obtained from GenBank database and included sequences of known actinobacterial PKS type II KS-domains. Two outgroups were included, *fabB* (beta-ketoacyl-ACP synthase I) from *Escherichia coli* involved in fatty acid synthesis and a PKS type II from *Photorhabdus luminescens* TTO1 (γ-Proteobacteria). When looking at the phylogenetic tree eleven groups could be differentiated on the basis of 95% similarity of the protein sequences. Sequences obtained from the clone library were very diverse as seen by the wide distribution of the different groups throughout the tree. Eight of the eleven groups clustered alone or close to known antibiotics producing PKS type II KS-domains (groups 1–7 and 11), while three groups clustered in close vicinity to known spore pigment producing KS-domains (groups 8, 9 and 10).

## Discussion

### Structural diversity of actinobacterial communities in the rhizosphere of beech

Using T-RFLP analysis of 16S rRNA gene fragments as a culture independent method to monitor changes in the actinobacterial beech rhizosphere community, the overall variability observed between different samples was very low. For all major peaks detected, the differences observed were merely on the level of peak intensities and no differences could be seen based on the presence or absence of these peaks. It can therefore be concluded that none of the applied factors (O_3_ and season) had the capability of qualitatively changing the actinobacterial community concerning its major components and that the general influence of the factors applied could be considered small. Nevertheless quantitative differences between samples for some T-RFs could be observed and clearly assigned to the influence of certain factors.

The clearest separation of samples was detected to be caused by seasonal shifts. Unique profiles were observed for spring samples and the major T-RF 102 bp responsible for this separation could be assigned to represent genera from the suborder Catenulisporinae based on comparisons with the clone library. This suborder contains mycelium-forming Actinobacteria, which are globally distributed and often isolated from acidic soils (Busti et al., 2006a,b; Tamura et al., 2008). Interestingly, no study documenting details of their ecology has been published so far and therefore the seasonal variability and dominance of this group observed in this study is a first indication of their active role for nutrient mobilization in forest soils. However, since the same peak was observed for two different genera (e.g., *Catenulispora* and *Actinospica* for T-RF 102 bp) the observed shifts cannot be assigned to a certain species. The phenomenon that several species or even genera are represented by the same peak in T-RFLP and other fingerprinting techniques has been reported in many studies (e.g., Smalla et al., 2007), thus interpretations of the results have to be done in general cautiously.

The drastic increase of T-RF 102 bp from spring to summer was statistically significant and similar for ozone treated and control plants. Seasonal shifts of microbial rhizosphere communities have been demonstrated in several studies (Smalla et al., 2001; Thirup et al., 2001) and in some cases these responses were associated specifically with Actinobacteria. Smalla et al. (2001) recorded a strong seasonal shift at the beginning of the vegetation period for rhizosphere communities of strawberries, oil seed rape and potato plants. Based on DGGE analysis of the rhizosphere communities they found indications that the abundances of bacterial high G + C populations were different during the developmental stages of all plants studied. Successional changes have also been described for plant associated Actinobacteria based on CFU counts and quantitative PCR methods (Thirup et al., 2001). The authors could show that at later time points in the season the abundance of Actinobacteria in the vicinity of barley roots increased significantly. They concluded that Actinobacteria are persistent during microbial succession beyond the early stages of root growth in annual plants due to their capability to penetrate and solubilize dead root litter (Thirup et al., 2001). Additionally, the active role of Actinobacteria in rhizospheres of diverse plants has been demonstrated in numerous studies (Billings and Ziegler, 2005). In the case of European beech the process of decomposition of old roots is likely to be more constant throughout the growing season compared to annual plants. The seasonal effect observed can thus not be assigned to increasing decomposition processes throughout the year. Additionally, the T-RF in question (T-RF 102 bp) peaked in summer and exhibited a slight decrease toward the end of the growing season. If this part of the bacterial population was mostly dependent on dead root litter as opposed to root exudates, it would rather be expected to peak in autumn, when the balance between new fine root production and older dead roots is largely in favor of the latter (Hertel and Leuschner, 2002).

A second statistically significant separation was observed in summer, where ozone samples showed unique profiles due to the relative reduction of one major T-RF (579 bp). Yet, the effect was very subtle and in this case transient, since autumn harvest of ambient and O_3_ treatments could not be differentiated any more. Clones from the library possibly representing this peak could not be identified by the RDP classifier. Therefore, the identity and ecology of this putative O_3_ responsive group would be of great interest and efforts are being made to isolate corresponding organisms from the studied soil. Only then, their functionality within the soil and possible implications on suppression of soil borne diseases can be investigated.

Responses of the microbial community to elevation of tropospheric ozone have been shown in other studies, but as observed in this study, the effects were relatively small in most cases. Dohrmann and Tebbe (2005) reported that from neither general Bacteria nor group specific SSCP profiles an ozone effect could be seen for the rhizosphere communities of different ozone-sensitive and insensitive herbaceous plants. The only exception in this study was the ozone sensitive composite *Sonchus asper* L., where changes were observed exclusively for the Actinobacteria specific profiles under elevated O_3_. In other studies effects on the fungal rhizosphere community were shown. Chung et al. (2006) demonstrated that elevation of O_3_ significantly altered the fungal community composition in a free-air enrichment experiment under three deciduous tree species utilizing DGGE. They also observed an increase in fungal relative abundance by PLFA analysis. In contrast to these result, Phillips et al. (2002) observed a decrease in fungal PLFAs while the relative proportion of Gram-positive and Gram-negative indicator PLFAs were not affected.

### PKS type II

This is the first study performed to investigate the effect of elevated ozone on genes potentially responsible for antibiotics production in the rhizosphere. PKS type II systems are of great interest when investigating the antibiotics production potential of actinobacterial soil populations (Wawrik et al., 2007). This especially holds true since a large portion of the actinobacterial community in the studied soil belongs to the newly described suborder Catenulisporinae. For this new lineage of Actinobacteria, Busti et al. (2006a,b) described a high potential to produce secondary metabolites with a polyketide scaffold. This is a feature they share with members of Streptomyces and related genera. All strains analyzed by Busti et al. (2006a,b) belonging to this group yielded distinct bands when checked with specific primers for PKS type I and II. For one of the strains, the production of a bioactive molecule similar to the well-studied antibiotic actinorhodin was demonstrated. This antibiotic is synthesized by a PKS type II system (Hopwood, 1997). However, when analyzing the diversity of PKS type II genes, an effect was discovered for neither season, nor an influence of the ozone treatment.

Since PKS type II genes do not follow the trend observed for 16S rRNA genes, it can be concluded that phylotypes responsible for the observed changes (e.g., T-RF 102 bp) do either not contain similar PKS genes or they do not possess PKS type II genes at all, which is very unlikely for members of the Catenulisporinae but cannot be excluded. Anyhow it can be stated that in this case no correlation could be seen between phylogenetic trends and the genotypical trait PKS type II. This is in line with the findings of Metsä-Ketelä et al. (2002) who observed that the phylogenies of 16S rRNA genes and PKS genes in Actinobacteria soil isolates were not congruent. They concluded therefore that the phylogenetic grouping of Actinobacteria is an inadequate predictor for the type of secondary metabolites they produce.

However, another explanation for a lack of a significant separation of PKS type II genes might be the high background noise of these fingerprints.. Almost 10% of all clones in the PKS type II clone library have to be considered “junk” DNA (e.g., pseudogenes and sequences with no homology). Their distribution is likely to be random since no selective pressure takes effect on them. Previous studies using this primer pair used presence-absence data rather than relative peak intensities (Wawrik et al., 2005). Yet, it is very likely that this will only increase the influence of minor peaks, since they would have the same impact on multivariate statistical analysis as major, well-reproducible peaks. Furthermore, an additional nine sequences (16.7%) were closely related to spore pigment producing genes. The necessity to differentiate between antibiotics and spore pigments producing PKS systems has been stressed by Metsä-Ketelä et al. (1999) due to different ecological functions of the resulting molecules. While the property of a molecule to act as a pigment does not yield any information about its chemical properties concerning bioactivity, a major difference is that spore pigments are presumably covalently attached to macromolecular components of spores (Lee et al., 2005). Furthermore precursors of pigments e.g., monomers could well act as antibiotics or have other functions, while their massive production leads to incorporation in pigment macromolecules (Kämpfer, 2006). Therefore, they might act as protective against grazing by microfauna of the soil, but are not very likely to be involved in antibiosis against competing microorganisms.

## Conclusion

On the basis of the performed analysis, the diversity of Actinobacteria possessing type II PKS genes was only slightly affected by seasonal changes. The applied ozone treatment did not have any effect on the distribution of these genes in the rhizosphere of beeches, although the ecophysiology of the plant was changed in response to the increased ozone levels in the atmosphere. Also the dynamics of the total phylum of Actinobacteria which were monitored in the presence study based on 16S rRNA gene fingerprints, was lower toward changes in the rhizosphere compared to other phylogenetic groups of bacteria, which showed significant changes in response to changes in the plant performance (e.g., bacteria of the genus Pseudomonas). This the large flexibility of Actinobacteria might be related their relatively large genome size and the possibility of this group of bacteria maybe to adapt faster and better to changing environmental conditions. Vice versa for the plant this indicates a huge stability of one important functional group of bacteria at the plant soil interface even if plant performance is changed. However, it has to be kept in mind, that changes were analyzed exclusively on DNA and not RNA levels. A transcriptomic approach might therefore yield further insights into the active part of the actinobacterial population. Furthermore the observed response pattern of Actinobacteria might change if beech trees of different age classes are studied or other stressors are investigated like increased drought periods or lack of nutrients.

## Author contribution

Felix Haesler, Alexandra Hagn, Marion Engel, and Michael Schloter were involved in the development of this study, in the planning of the experimental setup, data analysis and manuscript writing. Felix Haesler performed all experiments described in this study.

### Conflict of interest statement

The authors whose names are listed immediately below certify that they have NO affiliations with or involvement in any organization or entity with any financial interest (such as honoraria; educational grants; participation in speakers' bureaus; membership, employment, consultancies, stock ownership, or other equity interest; and expert testimony or patent-licensing arrangements), or non-financial interest (such as personal or professional relationships, affiliations, knowledge or beliefs) in the subject matter or materials discussed in this manuscript.
